# The Impact of Fluid Flow on Microbial Growth and Distribution in Food Processing Systems

**DOI:** 10.3390/foods14030401

**Published:** 2025-01-26

**Authors:** Zainab Talib Al-Sharify, Shahad Zuhair Al-Najjar, Zainab A. Naser, Zinah Amer Idrees Alsherfy, Helen Onyeaka

**Affiliations:** 1 Department of Oil and Gas Refining Engineering, Al Hikma University College, Baghdad 10052, Iraq; zta011@alumni.bham.ac.uk; 2 School of Chemical Engineering, University of Birmingham, Edgbaston, Birmingham B15 2TT, UK; 3 Department of Environmental Engineering, College of Engineering, Mustansiriyah University, Baghdad 10047, Iraq; zainab.naser@uomustansiriyah.edu.iq; 4 Chemical Engineering Department, College of Engineering, Al-Nahrain University, Baghdad 10081, Iraq; shahad.z.atta@ced.nahrainuniv.edu.iq; 5 Department of Quality Assurance, Northern Technical University, Mosul 41001, Iraq; zena.alshrefy@ntu.edu.iq

**Keywords:** fluid flow dynamics, microbial growth, food processing, food safety, turbulent fluid flow, laminar fluid flow

## Abstract

This article examines the impact of fluid flow dynamics on microbial growth, distribution, and control within food processing systems. Fluid flows, specifically laminar and turbulent flows, significantly influence microbial behaviors, such as biofilm development and microbial adhesion. Laminar flow is highly conducive to biofilm formation and microbial attachment because the flow is smooth and steady. This smooth flow makes it much more difficult to sterilize the surface. Turbulent flow, however, due to its chaotic motion and the shear forces that are present, inhibits microbial growth because it disrupts attachment; however, it also has the potential to contaminate surfaces by dispersing microorganisms. Computational fluid dynamics (CFD) is highlighted as an essential component for food processors to predict fluid movement and enhance numerous fluid-dependent operations, including mixing, cooling, spray drying, and heat transfer. This analysis underscores the significance of fluid dynamics in controlling microbial hazards in food settings, and it discusses some interventions, such as antimicrobial surface treatments and properly designed equipment. Each process step from mixing to cooling, which influences heat transfer and microbial control by ensuring uniform heat distribution and optimizing heat removal, presents unique fluid flow requirements affecting microbial distribution, biofilm formation, and contamination control. Food processors can improve microbial management and enhance product safety by adjusting flow rates, types, and equipment configurations. This article helps provide an understanding of fluid–microbe interactions and offers actionable insights to advance food processing practices, ensuring higher standards of food safety and quality control.

## 1. Introduction

Microbial control is an important aspect of food processing for the safety, quality, and shelf life of the product. Microorganisms are present in various places in the food production environment, and their presence affects the quality and quantity of the food product. Certain microbes are beneficial in fermented products as they contribute to and enhance the flavor, textural properties, and preservation [1,2]. Fluid flow dynamics is particularly crucial in the process of nutrient and gas distribution, and it is crucial for the metabolic activities of the fermenting microorganisms. The fluid movement ensures the appropriate diffusion of microbes throughout the product, resulting in a uniform fermentation and production of optimal organic acids, alcohols, and esters, among others that contribute to flavor and preservation. Furthermore, effective liquid flow sustains appropriate temperatures and oxygen levels, which are necessary for the growth of beneficial strains to ensure the quality and safety of the final product [3]. Nevertheless, in other instances, the presence of such microbes would mean spoilage, quality deterioration, and in some cases, exposure to health risks through food illnesses [4]. In this regard, food manufacturers have to control the amount and the location of the microorganisms, to comply with the legal requirements and consumer demands. Knowledge of the factors within these environments enables processors to make choices where the quality and safety of the product are at risk [5].

One such factor is food fluid flow which refers to the motion of liquids, such as that occurring during mixing, moving, or even storage. The different types of fluid flows create certain conditions that favor or limit microbial growth and dissemination within the system [6].

Conversely, laminar flows, which are described as smooth, sequential, and concentric flow layers along the surfaces, allow some microorganisms to settle and develop a biofilm, as seen in liquid storage tanks. These biofilms are protective coats of microorganisms that help them endure extreme environments, thus complicating the sanitation process. In addition to protecting harmful microorganisms, biofilms also play a crucial role in beneficial microorganisms, particularly in the process of fermentation. Biofilm formation can enhance the stability and functionality of these microorganisms, facilitating more efficient fermentation processes. In contrast, turbulent flow, such as in spray drying systems, disrupts biofilm development, posing different microbial control challenges [7,8]. Gaining insights into the intricate details regarding the fluid flow patterns and their effects on microorganisms is a key factor in controlling contamination risk.

Although the significance of fluid dynamics in microbial control is well acknowledged, there are various challenges that research in this area encounters. For instance, there is limited understanding of how changes in flow patterns affect the emergence of antibiotic-resistant strains or the adherence of pathogenic organisms on food surfaces. It is similarly difficult to model fluid dynamics in complex food matrices, such as multi-phase systems comprising solids, liquids, and gases. Another area of concern is the extent to which laboratory findings scale up to real-world production environments, where throughput is high and processing continuous, given the added complexity this entails in terms of microbial behavior and fluid flow [2]. These gaps block the construction of a full-fledged control system for different food production environments. Furthermore, the interaction of fluid dynamics with modern technology, such as the development of automated and real-time monitoring systems, offers further problems for the maintenance of product quality and safety in production systems.

Understanding fluid dynamics is essential for food hygiene and industry practice since movements and distribution patterns of fluids in food processing areas will have a direct influence upon microbial actions and, hence, the quality and safety of food products. Fluid flow conditions in fermented food systems directly affect the availability of the nutrients, temperature control, and gas exchange necessary for the optimum growth of microorganisms. Inefficient fluid motion makes fermentation uneven, leading to variations in quality, spoilage, or the proliferation of pathogenic microorganisms that may threaten health. Fluid velocity, mixing patterns, and temperature gradients contribute to the survival and growth of beneficial and harmful microorganisms. It is, therefore, important to understand how fluid flow interacts with microbial systems to design effective processing systems with minimal contamination potential, safety, and consistency of products [4]. This article analyzes the interrelations of microbial systems and fluid flow within the food processing environments, particularly in fermented food systems, where microbial growth is allowed and promoted. It aims to identify the varying impacts of fluid dynamics on the spread, growth, and exposure of microorganisms to hazards in food, to develop control considerations appropriate for the respective food production systems. This article is geared towards providing food processing industries with a thorough knowledge and understanding of fluid and microbial dynamics and how to establish appropriate systems that improve the quality and safety of the product through the proper management of microflora.

## 2. Fluid Dynamics in Food Processing Environments

### 2.1. Types of Fluid Flow

#### 2.1.1. Laminar Flow

Laminar flows are nearly always considered for fluids with high viscosities. In other words, driven by typical energy input, a fluid of this type in laminar flow mode is characterized by a viscosity index that is equal to or above 10 Pas. Moreover, due to the high viscosity of such fluids, their rheology is often highly intricate [9]. This flow regime usually prevails whenever it is complicated to agitate the large volume of high-viscosity fluid; however, this is the case most of the time. For instance, high-viscosity fluids can be observed in creams, pastes, and paints [10]. In laminar flows, the inertial forces tend to dissipate fast. This is normally the case, due to the presence of high viscosities in such situations. Therefore, it is more advisable to ensure that the rotating impellers occupy a large percentage of the vessel area to provide sufficient movement [11].

#### 2.1.2. Turbulent Flow

The fluid flow is referred to as turbulent in mixed vessels when the viscosity is less than 10 Pas. The central feature of all impeller designs is the movable impeller’s ability to induce fluid motion within the vessel; in turn, fluid is drawn toward and circulated back to the impeller. During this movement, high-velocity induced currents promote turbulent eddy diffusion in a circumscribing manner [12]. In turbulent flow, eddy diffusion enhances the process of mixing. This mixing is much higher than that in the case of laminar flows. This is because laminar flows, as well as transitional regimes, do not undergo eddy diffusion, which is the primary reason for the mixing not being efficient. In addition, in the dominant velocity gradient within the liquid that causes twister mixings, there exists the motion of turbulent eddies. Therefore, a turbulent flow is a region of well-dispersed fluids within a vessel [13].

### 2.2. Fluid Flow Characteristics

The understanding of fluid flow characteristics is of utmost importance during the processing of food as it affects both the mobility and transfer of mass within the food component, thereby determining its quality and consistency. In this connection, the technological operation of food production tends to control these flow properties since they influence processes such as dispersion, liquid transfer, and even heat exchange [14]. Flow characteristics for laminar flows depend on the properties of the liquids, the flow rate, and the sizes of the liquid and solid interfaces. With the rise in the mass flow rate, the momentum, or inertial forces, grow; but these forces are counteracted in the flowing liquid by frictional or viscous forces [6]. When these opposing forces are balanced at a specific adjustment, alterations in the flow characteristics begin to take place.

In addition to this, a concept employed in the analysis of fluid flow and its features in each of its branches is the Reynolds number, which is shortened to Re. This number, which is without a dimension, is the ratio of the effects of inertial forces (due to their movement) to viscous forces (due to their resistance) [15]. The Reynolds number is the most appropriate means of quantitatively defining the region of flow of a fluid, along the length of a tube or across the surface of bodies of other shapes [16]. This number shows that the movement of fluid will be either laminar (smooth and orderly) or turbulent (well mixed) [17].

The Reynolds number is an order of magnitude estimate for the viscous dissipation of energy in a fluid. If viscous forces are the main factor responsible for energy loss, then the Reynolds number is low, showing that the flow is smooth and viscous [18]. If the Reynolds number is maintained at a constant value of less than or equal to 2100 then the flow characteristics are streamlined or laminar [19]. A Reynolds number of more than 4000 suggests turbulent flow, which implies that the effect of viscous forces on energy dissipation is minimal [20]. For instance, fluids such as water and thin liquids tend to flow with little resistance, and under some circumstances, they become turbulent to facilitate interactions. In contrast, high-viscosity materials, like creams and sauces, tend to possess more resistance to flow and therefore tend to flow in a more laminar manner. The design of the equipment, especially that which involves stirring or pumping, is engineered to fit these flow types [9].

Table 1 provides a detailed comparison of laminar flow and turbulent flow, which are two distinct types of fluid flow, based on their characteristics and primary applications in the industry and their Reynolds number.

**Comparative analysis of fluid dynamics in food processing sectors**

Fluid dynamics is essential in optimizing food processing operations across different sectors, impacting the efficiency, safety, and quality of the final product. Each sector utilizes fluid behavior in various ways, such as mixing, pumping, heat transfer, and sterilization, but faces unique challenges due to the specific nature of the products being processed.

Table 2 provides a comprehensive comparative analysis of the role of fluid dynamics across various food processing sectors, highlighting both the unique challenges and fluid-related issues faced in each sector. Fluid dynamics is integral in ensuring optimal mixing, pumping, heat transfer, filtration, and other critical processes. However, each sector, such as dairy, meat, beverage, bakery, fruit and vegetable, cereal, and confectionery processing, encounters sector-specific fluid flow challenges, such as viscosity variations, air incorporation, and contamination control. The table also outlines the tailored solutions employed, from specialized pumps and mixing systems to advanced heat exchangers and filtration techniques, which help to overcome these challenges and ensure product quality, efficiency, and food safety in each food processing environment.

### 2.3. Computational Fluid Dynamics (CFD) in Food Processing

Computational fluid dynamics (CFD) plays a vital role in the modeling and simulation of processes involving moving fluids in many areas, including the aerospace and automotive industries, as well as food technology [30]. This involves studying the motion of liquids and gases, the laws of which can be expressed through equations that can subsequently be implemented by computers using computer software converted into the programming language [31]. This approach is typically implemented in the processes by one expert who has a basic understanding of all the subjects, making the optimization keenly focused on safety, quality, and cost-effectiveness [32].

In the context of food processing, computational fluid dynamics can be utilized to simulate and optimize thermal and mechanical processes such as baking bread, roasting and cooling beef roasts, or artificial drying in spray-drying systems. This helps to improve the quality, safety, and efficiency of food processing plants [33]. Traditional experiments, limited as they are to a few places within a system (where, for instance, sensors and meters are placed), cannot offer anything close to the capabilities offered by CFD. CFD simulations, including fluid flow, temperature distribution, pressure, velocity profiles, and heat transfer, make it possible for researchers to examine performance within a given area, using variables such as many flows and thermal processes [34]. CFD has the advantage of being able to run several different process scenarios, and also to focus on certain aspects, thus allowing for great control of how the processes work and aiding in research aimed at exploring a phenomenon without the expensive and prolonged laboratory investigation. This simulation-focused strategy enables design changes to be easily introduced at the beginning of process development, thus saving time and resources and increasing the scope for innovations in the use of fluid mechanics in all industries [35].

The study conducted by Habtegebriel et al. [36] applied a computational fluid dynamics (CFD) technique to simulate temperature profiles and their effect on the solubilization of milk proteins, such as αS1- and κ-caseins, during the spray drying of camel milk. With regard to outlet temperature deviations from real conditions, the CFD simulation predictions were 0.43% and 0.73% for the temperatures of 140 °C and 170 °C, respectively, with an 8.69% deviation for the temperature of 200 °C. The results revealed that the loss of protein solubility was related to the CFD-predicted temperature profiles, as confirmed by SDS-PAGE analysis. Furthermore, the influences of the drying temperatures on the physical properties of the resulting powder were explored, where higher temperatures above 160 °C were observed to exhibit an inversely proportional relationship between powder yield and moisture removal. SEM imaging disclosed rounder particles at high temperatures, while there were shriveled ones at lower temperatures, especially for cow milk. All these findings provide evidence that CFD in reality predicts important parameters involved in spray drying, and they provide insights into food processing optimization and recognition of quality parameters for simulation.

In Masud et al.’s [37] study, the simulation of a comprehensive transport model of a drying system using waste heat from engine exhaust was conducted. Small lab-scale IC engine exhaust reduces about 1137.15 kg of CO_2_ per year, which would otherwise be produced from a conventional food dryer of the same capacity. In addition, the very low cost of installation and the payback period make the drying system a viable economic solution. Furthermore, this system is also capable of reducing the considerable entropy.

To find the best thermal processing conditions for roasting goose breast meat, Szpicer et al. [38] relied on computational fluid dynamics (CFD) simulations, concentrating on protein denaturation and cooking loss. The research methods involved conjugate heat transfer and phase change models that included variations in meat composition (fat and water content) in the approach to enhance prediction accuracy. The optimum conditions outlined included 164.65 °C, 63.58 percent humidity, and a fan rotation of 16.59 rpm for 2000 s. The predicted levels of denaturation in myosin, collagen, and actin, as well as the cooking loss, correlated well with the experimental results. These findings thus prove that CFD is relevant in assessing protein denaturation and cooking losses in goose breast meat, thereby possibly improving product quality and consistency in gastronomy and the entire meat industry. With the new optimization method, the efficiency in food production is improved and sensory characteristics are raised as far as physicochemical properties are concerned, and nutritional value is also improved, leading to consumer satisfaction and therefore competitiveness in the market.

### 2.4. Application of Fluid Flow in Food Processing

The processing of food is the result of a number of factors acting synchronously, such as fluid flow, growth, and distribution of microbes, as determined by the raw materials, processing equipment, and time of processing [38]. These factors have to be studied as dynamic entities, requiring a holistic approach for predicting and controlling microbe behavior. The characteristics of raw materials, such as viscosity, particle size, and moisture content, influence the flow of internal fluids and their distribution of microflora. Similarly, the processing equipment determines the specific flow patterns and mixing efficiencies that govern the dispersion of microflora. All of these combined processing parameters, such as time, duration, and temperature profiles, affect microbial growth and inactivation, which are crucial determinants for product safety and quality.

To manage these complex interactions, predictive models that simulate both fluid dynamics and microbial responses under different processing conditions are crucial. Such models are very useful for food processors to predict their microbial risks and apply appropriate targeting measures in the actual control of microorganisms. With the link becoming increasingly clearer between fluid flow and microbial dynamics, food manufacturers will be able to extend the operational parameters optimizing sanitation protocols while keeping product integrity and safety. In this context, integrated solutions for individual applications of fluid flow during food processes, such as mixing, cooling, spray drying, and heating exchanges, become highly relevant.

#### 2.4.1. Mixing

For a food manufacturing process, mixing is one of the essential processes needed to distribute the contents evenly in multi-component combinations and also to obtain uniform products. Mixing is therefore the controlled application of the principles of fluid flow in food processes to achieve an even dispersion of particles within the food [39]. For instance, Jiang et al. [40] proposed two models of high-pressure homogenizers, which highlighted the feeding pressure, the oil–water ratio, and temperature for the enhancement of the emulsion mixing process. This particular study draws attention to the importance of flow parameters when considering the effectiveness of mixing processes and points out that some physical contents, especially in emulsions containing emulsifiers and extreme mass and heat transfer mechanisms, affect the homogenization of emulsions. Also, in the study conducted by Ameur [41], which investigated non-Newtonian fluids in a cylindrical vessel without baffles, different types of impellers, e.g., V-cut and W-cut, were examined for their effect on the flow traces and power. This study demonstrates how varying the impeller design can enhance the flow of fluids and hence reduce the mixing time when dealing with viscous food fluids. Additionally, Ferretti et al. [42] analyzed batch vertical mixing systems, examining the influence of the position of the rotor, the viscosity of the liquid, and the aspect ratio during the mixing of liquid with solid food. The results show that in configurations with an off-center rotor, a blade rotation speed of 50 degrees per second (8.33 rpm) generates a velocity distribution within the fluid. In the optimal configuration (low aspect ratio and high-viscosity fluid), only 24% of nodes exceed 0.025 m/s, while in the least favorable configuration (high aspect ratio and high-viscosity fluid), this percentage decreases to 12.7%. In these setups, the rotor diameters are much smaller than those in configurations with a centered rotor, leading to a significantly lower power involvement and reduced energy supplied to the fluid. The findings proved that some of the particular geometrical and physical characteristics have an effective influence on the results of the mixing process, showing how particular design features can be utilized to enhance the fluid processing of food systems.

#### 2.4.2. Cooling

To preserve food, it is very important to work with the airflow in food processing plants, especially in firms dealing with refrigeration and freezing. Such differences in temperature dynamics are especially relevant in the design of refrigeration systems for specific goods like fish, meat, fruits, or vegetables when these systems are operated through the cold chain [43,44,45]. One of the major considerations in such systems is the choice of refrigerant and cooling appliances because they have a direct effect on the microenvironment of the food during its storage and transport [46]. Temperature—especially during freezing—is controlled in such a way as to minimize the effects on quality, as explained in Miller et al.’s [47] study on ice crystallization in the production of ice cream. This study demonstrated the effect of the influences caused by scraped-surface heat exchangers on the size and distribution of ice crystals, which are crucial to the quality of the product after freezing.

The analysis of flow patterns has played an equally important role in the development of the designs of many refrigeration and freezing devices, from household and commercial equipment to even more complex structures, like industrial freezers; this emphasizes their applicability at different levels of the food business (Söylemez et al., 2021; Narsaiah et al., 2021) [48,49]. When it comes to the food freezing systems, researchers have since adopted various new ideas in process enhancement, such as hydrofluidization; in this case, some fluid flow at a given regime enhances the temperature distribution of the food. In the research by Stebel et al. [50], a broad range of liquid mass flow rates, from 0.1 kg·s to 2.0 kg·s, was examined. As a result, the heat transfer coefficients ranged from 1000 W·m·K to 4500 W·m·K, depending on the fluid mass flow rate. While the liquid type had a minor impact on the heat transfer coefficient (HTC), it influenced the behavior of the food samples. The presence of food product suspension in the HF tank was observed only with the ethanol solution at a moderate mass flow rate. The results revealed that heat transfer was hardly affected by the refrigerant type, but the behavior of the food samples undergoing freezing was markedly changed. In the cold store segment of the business, controlling the airflow within the unit is essential for quality storage. The dried foods also need proper temperature and humidity control during storage to avoid spoilage as they are hygroscopic. Alvarenga et al. [51] asserted this in their study on how traditional sheep cheese matured when the optimal parameters for the stored cheese were met without introducing any detrimental changes to its microbiological and chemical properties.

#### 2.4.3. Spray Drying

Spray drying is a very important method of food processing, but it also demands proper fluid flow dynamics, especially at the stage of atomization, spray–air interaction, and separation of the product. The trend of conducting spray drying operations using simulation models has been on the rise in recent times due to the difficulties and high costs involved in the practical determination of certain parameters, such as airflow, temperature, size of particles, and moisture content, in arrangements with numerous industrial dryers [52]. The proper application of the heat and optimization of the set process parameters is paramount in yielding a food product with its nutrients intact. If the process is not properly controlled throughout the drying period, heat-sensitive interactants, like vitamins and polyunsaturated fatty acid, may be lost, and for the products that have high moisture content and are heat treated, such as infant milk powder, excessive thermal treatments can result in the formation of toxic substances, such as polycyclic aromatic hydrocarbons (PAHs).

Spray drying results in qualitative improvements of the product and saves a lot of operational costs in terms of energy consumption when the spray drying process is optimized. The modeling of spray drying has mainly relied on two methods: the Eulerian–Eulerian and Eulerian–Lagrangian approaches. In the Eulerian–Eulerian model, the dispersed phase is modeled as a continuous fluid, whereas in the Eulerian–Lagrangian method, the gas phase is solved by the Eulerian approach, giving computational fluid dynamics to the spray as discrete particles [53,54]. Benavides-Morán et al. [54] employed the Euler–Lagrange model to analyze the airflow pattern when guava juice was dried by spraying. This enabled the authors to derive the trajectory of the particles from the spray nozzle to the walls and to the exit of the drying chamber, as well as to examine particle size, temperature, and humidity within the system and other factors related to the thermodynamic cycle of drying, thus allowing the high level of drying efficiency to be evaluated.

#### 2.4.4. Heat Exchangers

The integration of heat exchangers into food processing systems enables the easy transfer of heat from one fluid to another using either direct or indirect contact. Direct heat exchangers involve two fluids meeting, where hot and cold streams are introduced directly, which may involve non-miscible fluids or solids within a liquid [30]. However, in the food industry, indirect heat exchangers, such as recuperators and regenerators, are usually favored to prevent any mixing of fluids. These types of heat exchangers perform the function of heat transfer between fluids separated by a wall, which is common in processes like food processing, especially in dairy and juices where the quality and safety of the product are paramount [55].

In the dairy industry, especially in the processing and storage of milk, plate heat exchangers are used to pasteurize or heat the milk by making it flow in a thin layer between ‘hot’ plates. In essence, the proper temperature is maintained without milk being in contact with the heating medium. This method reduces fouling and incorporates a simple cleaning mechanism; hence, it is favored in the restaurant food processing operations that are continuous [56]. Lazaar et al. [56] illustrated that there were systems that could be properly utilized in small-scale dairy processing plants; these systems were localized and used plate heat exchangers incorporated with a solar water collector and absorption chiller. They managed to minimize the losses of milk spoilage in its deferment transport by adjusting fluid flow and temperature, which achieved an efficiency of 80% when milk was supplied at a temperature of 80 and a flow rate of 386 l/h. Furthermore, Sannad et al. [57] also focused on how fouling occurred during the milk pasteurization process. They reported that an appropriate arrangement of plates without deflector elements between them enhanced the efficiency of heat transfer, which is vital for controlling pasteurization results.

In the production of olive oil, tubular heat exchangers are employed to preheat the olive paste before oil extraction. Perone et al. [58] assessed this technique, where the olive paste is passed through the inner tube and water is supplied in the annulus outer jacket to heat the paste. This tube in a tube makes it possible to heat the olive paste to the appropriate temperature, enhancing oil extraction efficiency and quality during the heating of the paste. As fluid flow and temperature profiles are controlled, this system allows a high-quality processed olive with a very low amount of solids, which highlights the importance of fluid flow in food processing system design.

**Figure 1:**
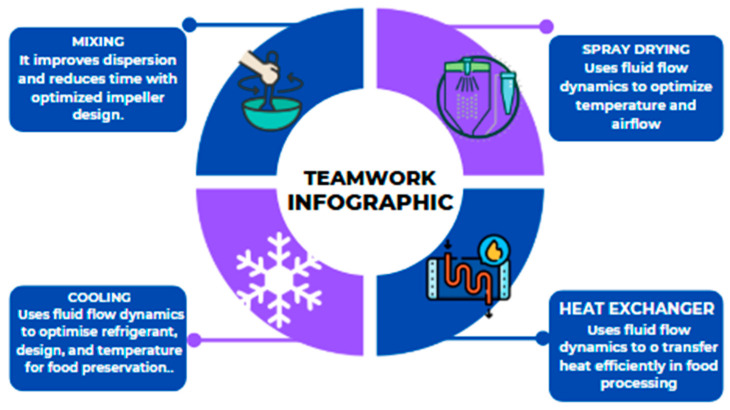
Applications of fluid flow in food processing.

Figure 1 illustrates key applications of fluid flow dynamics in food processing.

## 3. Factors Influencing Microbial Growth and Biofilm Formation in Fluid Environments of Food Processing Systems

### 3.1. Factors Affecting Microbial Growth

#### 3.1.1. Temperature

Temperature is one of the essential abiotic factors in microbial growth, particularly the growth rate and survival of the microorganisms. A particular range of temperature enables the growth of a specific range of microorganisms within that area, as growth below or above a particular temperature renders the organisms dormant or destroys them. More or less every organism has an optimum temperature which is conducive for growth. Low temperatures can slow down the growth of most microorganisms, while an increase in temperature above their optimum can successfully inhibit mesophiles while activating thermophiles [59]. In nature, the temperature range for the growth of the microorganisms is comparatively smaller than the one for the anthropogenic impacts, thus implying that there is an increase in the intra-specific competition of such factors as edibility or carrying capacity among the different microbial populations, and poorly adaptive taxa become extinct when the unfavorable outside temperatures persist more than the optimum ones in their lifecycles [60]. Some temperatures completely inhibit the growths of typical mesophiles and even prevent their existence, but these are the conditions suitable for hyperthermophiles [61]. In the same way, halophiles are found in saline habitats with high salt concentrations, but they do not thrive if put in neutral freshwater; non-halophiles are incapable of existing in salterns. Most living organisms develop at room temperatures (approximately 18–20 degrees Celsius) and while temperature extremes can be used by fungi in their growth and reproduction, such temperatures are not without adverse effects on growth [49]. Moreover, the temperature change is an important critical factor when dealing with food products and their storage to avoid the growth of microorganisms, as it is known that the majority of the food-borne pathogens grow optimally between 20 degrees and 45 degrees centigrade; however, psychrophilic bacteria grow optimally at lower temperatures, while thermophiles grow at relatively high temperatures [62]. The ability of temperature changes to interfere with a population of microorganisms may alter the rate of spoilage and the composition of the microorganisms in a food product, thus highlighting the role of temperature control in food safety [63].

#### 3.1.2. Oxygen Levels

Microorganisms exhibit a wide range of oxygen needs. Aerobic microorganisms cannot grow without oxygen, while anaerobic organisms flourish without it. However, facultative anaerobes are those that can live with or without oxygen as per the environmental conditions. In fluids, flow rates and agitation eliminate boundaries and facilitate a compromise between oxygen diffusion and microbial growth [64]. As an example, some aerobic microorganisms benefited from excess flow rates where increased oxygen transfer favored their growth. However, in operable conditions or low flow regions, the negative flow dynamics in the enzyme conditions could support the development of pathogenic organisms, which explains the need for careful equipment management and application to ensure that the oxygen limits are achieved [65].

#### 3.1.3. pH

The pH of a fluid environment also plays an important role in determining whether microorganisms survive and grow or not [66]. Most bacteria grow best in a pH range of about 7 to 7.5, which is neutral to slightly alkaline, while fungi prefer a pH of between 5.2 and 5.6, which is acidic. Molds have a wider pH range and are not that affected by fluctuations in pH; however, they grow best at about pH 4 to 4.5 [67]. Regulating pH levels can also be vital for food industries in their processing since it can hinder the growth of epidemic organisms, particularly in pickles and other fermented products where the use of acids is advocated. Furthermore, changing the pH of a system can also vary the activity of the metabolic enzymes in microorganisms or, in turn, other physiological processes, thereby enhancing or lowering the quality and safety of food products [68].

#### 3.1.4. Nutrient Availability

The development of microbes is influenced by the presence of limiting macronutrients such as carbon and nitrogen, micronutrients such as vitamins and trace elements, and other essential nutrients. Fluid food processes often operate with nutritional level variations which may help either lower or raise the level of microbial activities and growth [69]. For instance, nutrient-dense media may promote rapid colonization by microbes, leading to the risk of spoilage; hence, food safety measures are put in place. In addition, the nutrient availability might also depend on the presence of other microorganisms since certain species might dominate while others are suppressed due to resource competition within the population, which will change the composition of the microbial community as a whole [70]. Differential flow rates converting into differential nutrient distributions lead to a diversity of microhabitats within biofilms. Nutrient limitation under slow-flow conditions enhances competition among different species and their spatial organization and growth dynamics. Conversely, the increased flow rate increases nutrient supply, which gives rise to more synergistic interactions and higher biomass [71].

### 3.2. Influence of Fluid Flow on Biofilm Formation

Biofilms consist of certain groups of bacterial cells, such as *S. aureus*, *S. epidermidis*, *E. coli*, *P. aeruginosa*, and *K. pneumoniae*, adhering together through a glue-like matrix of extracellular polymeric substances (EPSs) [72]. Biofilms are commonly found in various environments, including stored food processing vessels, such as fermentation tanks, storage silos, and pasteurization systems, where they can impact food quality and safety [73]. They can also be found in rivers [74], coastal regions, drinking water distribution systems (DWDS), and many organs inside the human body. Biofilms have also been employed in environmental bioengineering; for instance, these layers have been used in washing and even suppressing polycyclic aromatic hydrocarbons (PAHs), oil enhancement, and the treatment of wastewaters by excess nutrient shedders [75]. Biofilms develop in an environment of turbulent flow where the Reynolds number (Re) exceeds 5000. Biofilm development is more rapid in a turbulent environment [76].

In an environment where microbiologically influenced corrosion (MIC) occurs within an engineered metal or tubing system, the biofilm growing on its metallic surface affects the activities of the metal surfaces, that is, the cathodic and/or anodic reactions or surface characteristics, thus inhibiting or promoting the corrosion behavior [76]. For instance, in dairy processing, the constant flow of milk through pipelines can lead to biofilm formation, which, over time, promotes MIC. Similarly, in beverage production, the movement of liquids through metal pipes can facilitate the growth of microbial biofilms, increasing the risk of corrosion, especially in high-humidity environments. Biofilm formation is a never-ending process, characterized by controlled movement; it is also complicated and is affected by a multitude of things, such as the organisms involved, the type of surface of the metal, and the surrounding environment, to mention but a few [77]. In pipelines, the flow discharge of the transported fluid is one of the essential parameters that governs the biofilm development and its morphology. It was hypothesized that MIC is more likely to manifest itself with still water. Studies suggested, however, that microbiological corrosion might also take place in a moving liquid [78].

In addition, cell distribution in the flow interior of pipes is also influenced by fluid motion. The wall shear stress, which is a function of the flow velocity, minimizes the risk of biofilm detachment from the steel substrate [79]. A study by Liu et al. [80] found that at a higher flow velocity of 4.17 mm/s and a reduced nutrient concentration of 1 mM, the effect of biofilm development in a porous medium was proven to produce very little formation, as a result of the shear forces experienced by the biofilm in the porous medium; additionally, the nutrient level in the bioreactor was not significant enough to sustain the growth of the microorganisms, thereby creating a ‘no/low growth region’. Therefore, when the flow velocity was reduced to 1.66 mm/s, and the concentrated nutrient medium was increased to 10 mM, biofilm coverage increased rapidly and showed considerable strength against shear forces. The formation of biofilms was generally inhibited with excessive shear stresses, while it favored growth with high nutrient levels; however, weakened attachment was observed because of high nutrient levels. This allowed cells to become more susceptible to detachment. Additionally, a study by Intan and Santosa [81] showed that, under the laminar flow conditions with Reynolds numbers (NRe) of less than 2000, the rate of average biofilm formation was around 0.32 mm/week, which indicated a quite low rate of biofilm growth. On the other hand, under the turbulent flow conditions with NRe greater than 3000, biofilm formation was about 0.53 mm/week, which suggested an enhancement of biofilm deposits due to turbulence.

Fluid motion in pipes positively influences mass transfer and creates a wall shear stress that inhibits cell attachment. It should also be noted, however, that fluid flow facilitates a loss of previously established biofilm. The suspension of a pre-existing biofilm of *Pseudomonas aeruginosa* grown under laboratory conditions in a flow cell was proven possible [82] and occurred upon application of a stream of liquid [83]. Liu et al. [84] studied the factors affecting biofilm development and microbiologically influenced corrosion (MIC) on X70 pipeline steel in the presence of sulfate-reducing bacteria (SRB) in oilfield water at multiple flow velocities. It was revealed that at a lower flow velocity (0.2 m/s) biofilm developed, resulting in extensive MIC and pitting-type corrosion on the surface of the steel. In contrast, increasing flow velocity to 1.0 m/s inhibited biofilm development and thus led to a decrease in MIC. At low flow conditions, both the corrosion products and biofilm were present on the steel surface, while at a higher flow, the layer was predominantly made of corrosion products with less biotic activity.

## 4. Microbial Adaptations to Flow Condition

Microorganisms can quickly adjust to changes in fluid flow, especially shear stress and flow patterns (Table 3). Such adjustments are important for their survival and proliferation in colonizing new places, whether in natural environments or industrial activities like food or wastewater management [85]. The ability of these organisms to exist and flourish in various flows further emphasizes their ecological and industrial relevance [86].

### 4.1. Resilience to Shear Stress

Microorganisms have different physiological and morphogenetic aspects that assist them in withstanding the sheer stress induced by flow in a moving fluid [91]. For example, many bacteria have developed features to help prevent desiccation, such as thickened cell envelopes, the formation of capsules, or structures such as pili and fimbriae. These features promote adhesion to the surfaces and protect the tissues from shear stress [92]. These structural features not only assist microbes’ resistance to the physical stresses of liquid flow but also promote the production of biofilms, which protect them even further from unfavorable environments [93]. In addition to structural variations, other forms of motility mechanisms constitute an important aspect of microbial resilience. For example, some microorganisms, such as some species of *Pseudomonas* and *Escherichia coli*, have flagella, which assist them in swimming against fluid currents. This kind of motility enables them to move towards a positive place of growth, avoiding all the non-ideal regions, and it allows better adherence to surfaces. Their capacity to alternate between motile and sessile states reinforces their strength in a variable flow environment, enabling them to be more competitive in such scenarios [94].

Microbial species show structural modifications to withstand shear stress, assuring their survival and persistence under duress. For example, *Escherichia coli* can withstand a high shear force by relaxing stress across a very broad range of hydrodynamic conditions, keeping its cell wall intact and the structure stable under mechanical stress [95]. *Listeria monocytogenes* is usually found in food processing systems, and it overcomes shear force effects by modifying its membrane lipid concentration of unsaturated fatty acids, so that under stress conditions, it has increased fluidity and membrane integrity; such adaptations increase resilience to washing systems and high-shear food processing equipment [87]. Additionally, *Pseudomonas aeruginosa* forms very strong biofilms, which not only resist being washed out but also prolong the adhesion time under shear; thus, it develops well on the surfaces in dynamic flow environments, like dairy pipelines and beverage production systems [88].

### 4.2. Responses to Flow-Related Factors

Microorganisms may also adjust to other flow-related parameters, such as the concentration of nutrients, the level of turbulence, or the properties of the fluid itself. In a fluid motion, e.g., fluid flow, the availability of nutrients can change because of the action of the fluids. All these adaptations help microorganisms to cope with the high level of competition and even keep up their growth kinetics in a medium with varying nutrient concentrations [85].

Under turbulent flow conditions, microbes may find it difficult to exist owing to the high shear forces and the risk of starvation. On the other hand, some microorganisms manage to survive in those conditions by altering their metabolism to use other energy sources or by creating biofilms that can resist turbulence. For instance, *Bacillus subtilis* can produce very thick biofilms that are capable of withstanding turbulent flows with active metabolism, indicating that this organism goes beyond ordinary environments [89].

Metabolic flexibility, in particular, is a significant adaptation among microorganisms residing in fluid environments. It is common for many organisms to vary their metabolism in response to the substrates that are present and the flow conditions. For example, certain bacteria can utilize aerobic and anaerobic modes of respiration based on the availability of oxygen in the surroundings. This capability for metabolism is a great benefit in such an environment as theirs, where flow regimes, especially, are quite different [90].

## 5. Impact of Fluid Flow on Microbial Contamination and Distribution

### 5.1. Microbial Spread in Turbulent vs. Laminar Flow

Regarding microbial spread, fluid flow plays an important role, with its most fundamental aspect being the ease of the flow. For instance, the microbial spread is even more pronounced in turbulent flow, which is marked by the irregular and chaotic movement of the liquid and the presence of eddies, than in laminar flow, which is a slow and steady movement of the fluid in parallel layers with the least disturbance. In such turbulent conditions, quicker agitation and movement of microorganisms within a system occurs [86]. This higher degree of mixing allows better transport of microorganisms, enabling them to access surfaces, as well as recesses, which would otherwise be difficult or impossible to reach [96]. The downside however is that the chances of contamination are high since microbial contaminants, such as pathogens, can also be contained in the fluid and transported to different points of a processing unit, making them difficult to control and eradicate. Turbulent flow in food production environments, such as in dairy processing, beverage production, and juice processing, can lead to contamination by disrupting microbial populations and causing uneven heat distribution or biofilm detachment [86]. In dairy processing, this can result in pathogens like *Listeria* or *Salmonella*; in beverage production, it can spread *Pseudomonas* species; and in juice processing, it can promote spoilage organisms [97].

On the other hand, the laminar flow approach restricts the migration of microorganisms, resulting in their distribution within a confined area. In such cases, stagnant flow region configuration can favor the growth of such organisms, leading to biofilm formation. In designs employing laminar flow, the threat of excessive contamination is minimal, but biofilm presence becomes an issue as they tend to be more resilient against cleaning and disinfecting methods. Biofilm formed in such conditions resists cleaning and disinfection, becomes a part of the contamination cycle, and reduces the overall efficiency of the system [85]. Hence, the laminar flow of fluids, as mentioned, does restrict microbial movement and can sometimes lead to increased concentrations with biofilm formation, restricted mixing and dispersion, and increased risk of contamination due to stagnant zones. To minimize these detrimental effects, food processors may opt for regular preventive maintenance cleaning schedules with proper sanitizer application, optimizing flow design through very slight turbulence to minimize stagnant zones, and monitoring microbial growth for causes and timely intervention using sensors or microbial sampling. Temperature control can also limit microbial growth as many pathogens grow at specific temperatures; in doing so, food processors minimize trade-offs and improve safety and efficiency in their systems. These flow dynamics are crucial in the development of effective plans and approaches for the control of microbial contamination [85].

### 5.2. Risks of Cross-Contamination

The dynamics of fluids in motion can enable cross-contamination. This entails the transfer of microorganisms from one surface or area to another. This is more worrying in places such as food processing in Rajkot or health care centers, where the issue of hygiene is taken very strong seriously. When fluid moves across surfaces, it carries away dirt which may include bacteria and viruses from the surface to another surface which may have been cleaned previously [98]. For instance, many vectors are associated with microorganisms when they are related to a surface immersed in a running fluid. The load of microorganisms could be transported downstream, thus increasing the chances of contaminating areas far away from the contaminated surface; it can also happen in other ways, such as by splashing or the movement of droplets within fluid [99]. Also, those droplets are end-attached microorganisms, and the free flow of water can enhance their transfer during a turbulent flow. Most of the time, when the fluids are disturbed or sprayed, very small drops are formed and thrown into the air which propagates the presence of diseases through the air. This is a more critical problem in places like hospitals and food service units where harmful microorganisms present a great risk. All these do not happen easily; this is because they require a lot of control over the processes of fluid movement and the prevention of contamination [100].

### 5.3. Contamination Control Challenge

When flow dynamics in a system have poor control, there is a greater risk of microbial contamination. This is attributed to several causes, including inadequate flow design, insufficient monitoring, and maintenance, as well as interactions of flow with microbial activity. After all, systems that do not take into account the efficient flow of fluids may have some areas where no internal circulation occurs, leading to an enhanced ability of microorganisms to flourish. Such areas of static fluids may lead to contamination which will be hard to eliminate during the cleaning process and will pose a risk of cross-contamination during the procedure [101]. Internal factors such as float flow and microbial population limit the risk of contamination. Additionally, if there is no proper upkeep and control of the system, the flow dynamics within the system may easily vary and further contribute to its contamination. Furthermore, fluid flow, surface dynamics, and biofilm behavior are not always easy to determine; thus, the patterns of how and where they contaminate differ widely. This knowledge is imperative in formulating effective interventions aimed at reducing the risk of contamination [102].

## 6. Strategies for Managing Fluid Flow to Control Microbial Activity

### 6.1. Design Modifications in Equipment

To control the microbial load in processing systems, changes in food processing equipment, such as flow rate modifications, pipe size adjustments, and mixing speed optimization, are necessary. Alteration of flow rates can enable fast or slow passage of liquids through processing machines, which affects the spread of microbes [103]. For example, smaller pipes tend to increase the speed, which decreases the chances of bacterial build-up, while in processes that require more even spreading, larger pipes control the flow to a steady state. Customizing mixing speed also helps in agitating the components at the right speed and helps in the reduction in any idle regions where microorganisms can hibernate and replicate. For instance, ultrasonic agitation has been shown to improve microbial reduction by creating high-frequency sound waves that enhance fluid mixing and disrupt biofilms, making it more difficult for microorganisms to proliferate. This technique has been successfully applied in dairy and beverage processing, where it helps reduce bacterial contamination without compromising product quality. It also makes it easier to produce hostile conditions to infection without reducing the productivity of the operations [104].

### 6.2. Flow Control for Enhanced Microbial Growth in Fermentation

Precession in the regulation of flow within a fermentation process is significant for the growth and performance of the desired organisms as the different products developed have specific flavors, textures, and safety levels, which require certain conditions to be present. Flow control has enhanced fermentation outcomes by ensuring uniform nutrient distribution and consistent microbial activity. In processes like yogurt or beer production, regulating flow rates and agitation prevents stagnation, leading to more efficient fermentation, better product consistency, and improved flavor profiles. It also helps maintain optimal temperature and nutrient gradients for faster fermentation [105]. Hydrodynamic features enable the regulation of temperature, oxygen, and nutrient addition, and these attractive conditions make it possible for the microorganisms to grow fast. For instance, a low Reynolds number turbulent flow is suitable for the growth of good bacteria in the production of fermented products such as yogurt, while a controlled amount of turbulence is appropriate for aerating drinks such as kombucha. This type of control not only improves the quality and uniformity of the fermentation products but also prevents the entry of pathogenic microbes to a level that will not compromise the degree of microbial activity necessary for fermentation processes.

### 6.3. Antimicrobial Surface Treatments and Flow Regulation

To reduce microbial adhesion and biofilms in processing systems, it is effective to combine antimicrobial surface treatments with controlled fluid flow. Processing equipment, such as pipes, tanks, valves, and surfaces, can be treated with antimicrobial coatings, e.g., silver or other safe-for-body usage materials to lessen hydrophilic conditions. If coupled with fluid residence time that is non-existent or has a low regime—regular movement of the system—these treatments are effective in impeding the development of biofilm, which, once formed, is very difficult to remove and is also a breeding ground for microorganisms [106]. The continuous movement of the fluid tends to remove a few of the organisms that are trying to settle down within the surface, and at the same time, the coating works in preventing the further settlement of the organisms. These techniques produce a cleaner processing area, which helps in reducing the levels of undesirable contaminants and consequently enforces stricter food safety measures [107].

Table 4 provides a concise overview of how fluid dynamics influence microbial activity and the measures needed to minimize contamination risks in these systems.

## 7. Implications for Food Safety and Quality Control

### 7.1. Risk Reduction in Industrial Settings

Grasping the fluid dynamics within the food processing systems has the potential to improve the safety measures that help control the negative aspects brought about by microorganisms. Optimum fluid flow management will ensure that there are no zones of stagnation created where pathogens are likely to settle, creating an atmosphere that is unwelcoming to harmful microorganisms that produce biofilms. The speed of fluid movement in food processing, such as in milk pasteurization, plays a crucial role in reducing contamination by improving heat transfer and preventing stagnant areas where microbes can grow. Optimizing flow rates and agitation enhances uniform heating, effectively killing pathogens and reducing microbial contamination. For example, higher fluid velocities in pasteurization systems improve heat penetration and safety, while also boosting system efficiency. Similar optimizations in the beverage industry have demonstrated reduced contamination risks and improved product safety and consistency.

With the knowledge gained from the study of fluid mechanics, relevant contamination control measures can be instituted in the processes aimed at industrialization. For example, it is possible that the incorporation of changes that manage the fluid movement speeds and how the tools are arranged can drastically reduce the areas that support the growth of disease-related germs, thereby improving the safety of the manufacturing process. In addition to this, the monitoring of fluid dynamics in real time can make it possible to respond to any threats of contamination within a short period and thus improve food safety measures even more.

### 7.2. Product Consistency and Quality Assurance

The optimization of product flow within the system of a food product being manufactured contributes to the even distribution and activity of the microorganisms within the entire process, thereby ensuring the same quality of the final product. In fermented products and other products which are to some extent dependent on microbes, content uniformity is very important as it affects the flavor, the mouthfeel, and the shelf life of the product. This control helps prevent spoilage from competing microbes, while selectively favoring the desired produce and therefore ensuring the acceptable quality of the products at all times. Adequate utilization of fluid mechanics leads to desirable and reproducible outcomes in batch production, which is imperative in ensuring customer loyalty and compliance with available laws in the region. In addition, better regulation of the location of the microorganisms leads to enhanced predictability of the fermentation and processing stages, thus improving the quality of the product and stabilizing the low-quality deviation.

### 7.3. Future Considerations

The exploration of how microorganisms interact with hydration media creates multiple research opportunities aimed at enhancing the processes involved in food safety and its preservation. Understanding the impact of different fluid dynamics on microbial behaviors would help create effective measures for the prevention of contamination, especially for high-risk products characterized by the need for utmost safety from harmful microbes. In the future, comprehensive research will aim at the design of complicated systems to control the flow of fluids in favor of the controlled manipulation of other living microbes to verify the safety and quality of the end product. Furthermore, the motion of fluids in different food systems should be studied to assist in understanding how to create favorable conditions for the good microbes in the system; this will help in creating more advanced food processing technologies. However, research gaps remain, particularly in the role of emerging technologies, such as AI-driven computational fluid dynamics (CFD) models. These models can play a pivotal role in predicting microbial behavior within fluid systems by simulating real-world scenarios and identifying conditions that favor or inhibit microbial growth. Such models have the potential to significantly improve the design and optimization of food processing systems, offering targeted solutions for controlling contamination. These strategies, driven by technological advancements, will enable the food sector to better control microorganisms, reduce contamination risks, and lower the chances of exceeding safe contaminant levels. These strategies will enable the food sector to enhance its control over microorganisms and therefore lower the chances of exceeding safe levels of contaminants.

## 8. Conclusions

In food processing systems, fluid flow is an important determinant of microbial growth, distribution, and control of contamination, which implies that the dynamics of fluid motion must be well managed for the safety and quality of food by minimizing chances of contamination and enhancing the activities of microorganisms which are beneficial. This article emphasizes that it is possible to optimize flow and to enable better control of microorganisms, less risk of product safety, and better consistency of the product; thus, fluid dynamics is also of critical importance in the process of increasing the efficiency of food processing and controlling its quality. To improve food safety and quality, future research should focus on the fluid–microbe interaction for more food systems and the advanced control of flow, which may in turn help in the development of safer and more effective food processing systems.

## Figures and Tables

**Table 1:** Turbulent and laminar fluid flow characteristics.

Flow Type	Characteristics	Reynolds Number	Applications	References
Laminar Flow	Occurs in high-viscosity fluids (viscosity index ≥ 10 Pas). Smooth and orderly fluid movement in parallel layers. Inertial forces dissipate quickly, with minimal turbulence. Requires large impeller area for movement.	Reynolds number ≤ 2100 (smooth, viscous flow)	Ideal for handling high-viscosity materials, like creams, pastes, and paints. In dairy processing, it is used for homogenizing milk and cream. In beverage production, it is applied in the mixing of concentrated juices and syrups.	[9,10,11]
Turbulent Flow	Occurs in low-viscosity fluids (viscosity < 10 Pas). Fluid movement is characterized by high velocity and turbulent eddy diffusion. Promotes efficient mixing and dispersion. Little resistance to flow due to low viscosity.	Reynolds number > 4000 (turbulent, well-mixed flow)	Suitable for low-viscosity fluids, such as water and thin liquids, ensuring efficient mixing and dispersion. In beverage production, it is commonly used in soft drink carbonation and beer brewing processes.	[12,13]

**Table 2:** Table showing comparative analysis of the role of fluid dynamics across various food processing sectors.

Food Processing Sector	Role of Fluid Dynamics	Challenges and Fluid Dynamics Issues	Solutions/Strategies	References
Dairy Processing	Mixing, pumping, heat transfer, and sterilization of dairy products.	The viscosity of milk changes with temperature, fat content, and additives, affecting pumping and mixing efficiency. Fluid flow can also contribute to microbial contamination. The need for precise temperature control during pasteurization poses a challenge in heat transfer.	Use of high-efficiency pumps and heat exchangers to maintain stable flow conditions. Implementation of hygienic pumps and pipe systems, along with pasteurization and sterilization. Adoption of counter-current heat exchangers for uniform heating.	[21,22,23]
Meat Processing	Emulsification, pumping, air incorporation, and cleaning processes within meat products.	The viscosity of meat products, like sausages and broths, can be higher, requiring specialized flow management. Variations in fat and water content affect flow behavior. Trapping air during processing can affect product texture. Complex cleaning requirements due to fat buildup and debris complicate sanitation.	Use of mixers and emulsifiers designed for high-viscosity products. Adjustment of pumping systems to accommodate fat and water content fluctuations. Use of vacuum systems to control air incorporation. Enhanced CIP systems using high-pressure fluids and cleaning agents.	[24]
Beverage Processing	Mixing, carbonation, filtration, and separation processes in beverage production.	Carbonation must be precisely controlled to avoid over- or under-carbonation. Achieving uniform mixing of concentrates, flavors, or syrups in large batches can be difficult. Filtration and separation processes are necessary to remove particulates or clarify the product.	Precision carbonation systems and degassing units. High-shear mixers, agitators, and homogenizers for consistent ingredient distribution. Filtration systems (e.g., membrane filtration) to achieve clarity.	[25]
Bakery Processing	Dough consistency, aeration, heat transfer, and coating applications.	Dough consistency, particularly in bread and pastries, varies and affects handling. Air incorporation in dough is crucial for desired texture, but it can be difficult to control. Uneven heat transfer during baking can result in inconsistent product quality. Fluid-based toppings and glazes are challenging to apply uniformly.	Automated dough handling systems and mixers for various dough consistencies. Controlled mixing techniques and air-extraction systems for aeration management. Convection ovens and optimized airflow systems for uniform heat distribution. Precision coating systems for fluid applications.	[26,27]
Fruit and Vegetable Processing	Aids in juicing, washing, filtration, and enzymatic control in fruit and vegetable processing.	High water content in fruits and vegetables affects fluid flow, especially during washing or juicing. Separating excess water from juices and purees while concentrating them can be challenging. Enzyme activity during processing can alter the consistency of the product, affecting flow.	Specialized juicers and washers designed for high water content. Membrane filtration or centrifugal separators for liquid concentration. Precise temperature and pH control to manage enzymatic activity.	[28]
Cereal Processing	Influences milling, extrusion, puffing, and moisture control in cereal processing.	Milling generates high shear forces, affecting the consistency of flour. The puffing and extrusion processes require careful management of air and fluid flow to achieve the desired texture. Controlling moisture levels in cereal products is essential to prevent spoilage and maintain texture.	Controlled milling systems to minimize fluid turbulence. Optimized extrusion pressure, temperature, and air flow for consistent puffing. Humidifiers and air dryers to regulate moisture during processing.	[29]

**Table 3:** Summary of microbial adaptation to flow conditions.

Adaptation Type	Description	Examples
Resilience to Shear Stress	Microorganisms develop structural features like thick cell envelopes, pili, and fimbriae to resist shear stress and promote adhesion. Flagella helps motility.	*Pseudomonas* and *Escherichia coli* use flagella to move against fluid currents [87,88].
Responses to Flow-Related Factors	Microorganisms adjust to changes in nutrient concentrations and fluid turbulence. They use transport proteins for nutrient uptake and form biofilms.	*Bacillus subtilis* produces thick biofilms to withstand turbulence [89].
Metabolic Flexibility	Microorganisms alter their metabolism based on available substrates and flow conditions, switching between aerobic and anaerobic respiration.	Adaptations in metabolism for varying flow regimes [90].

**Table 4:** Impact of fluid flow on microbial contamination and strategies for management.

Impact	Description	Strategies for Managing Fluid Flow
Microbial Spread in Turbulent vs. Laminar Flow	Turbulent Flow: Increases microbial spread due to chaotic movement. Laminar Flow: Limits migration but can promote biofilm formation.	Design Modifications: Adjust flow rates, pipe sizes, and mixing speeds to control spread and prevent biofilm.
Risks of Cross-Contamination	Fluid flow can carry microorganisms across surfaces, increasing contamination, especially in food processing and healthcare.	Fermentation Flow Control: Regulate flow in fermentation to enhance the growth of desired microorganisms and prevent pathogens.
Contamination Control Challenge	Poor flow control leads to stagnant areas where microorganisms thrive, complicating cleaning and contamination prevention.	Antimicrobial Surface Treatments: Use antimicrobial coatings with controlled flow to reduce microbial buildup and improve hygiene.

## Data Availability

No new data were created or analyzed in this study. Data sharing is not applicable to this article.
